# Resting state functional network switching rate is differently altered in bipolar disorder and major depressive disorder

**DOI:** 10.1002/hbm.25017

**Published:** 2020-05-13

**Authors:** Shaoqiang Han, Qian Cui, Xiao Wang, Liang Li, Di Li, Zongling He, Xiaonan Guo, Yun‐Shuang Fan, Jing Guo, Wei Sheng, Fengmei Lu, Huafu Chen

**Affiliations:** ^1^ The Clinical Hospital of Chengdu Brain Science Institute, School of Life Science and Technology, University of Electronic Science and Technology of China Chengdu China; ^2^ MOE Key Lab for Neuroinformation High‐Field Magnetic Resonance Brain Imaging Key Laboratory of Sichuan Province, University of Electronic Science and Technology of China Chengdu China; ^3^ School of Public Affairs and Administration, University of Electronic Science and Technology of China Chengdu China

**Keywords:** bipolar disorder, dynamic networks configuration, fMRI, major depressive disorder, multilayer network method, network switching rate

## Abstract

The clinical misdiagnosis ratio of bipolar disorder (BD) patients to major depressive disorder (MDD) patients is high. Recent findings hypothesize that the ability to flexibly recruit functional neural networks is differently altered in BD and MDD patients. This study aimed to explore distinct aberrance of network flexibility during dynamic networks configuration in BD and MDD patients. Resting state functional magnetic resonance imaging of 40 BD patients, 61 MDD patients, and 61 matched healthy controls were recruited. Dynamic functional connectivity matrices for each subject were constructed with a sliding window method. Then, network switching rate of each node was calculated and compared among the three groups. BD and MDD patients shared decreased network switching rate of regions including left precuneus, bilateral parahippocampal gyrus, and bilateral dorsal medial prefrontal cortex. Apart from these regions, MDD patients presented specially decreased network switching rate in the bilateral anterior insula, left amygdala, and left striatum. Taken together, BD and MDD patients shared decreased network switching rate of key hubs in default mode network and MDD patients presented specially decreased switching rate in salience network and striatum. We found shared and distinct aberrance of network flexibility which revealed altered adaptive functions during dynamic networks configuration of BD and MDD.

## Graphical abstract

This study explored distinct aberrance of network flexibility during dynamic networks configuration in BD and MDD patients. We found shared and distinct aberrance of network flexibility which revealed altered adaptive functions during dynamic networks configuration of BD and MDD.

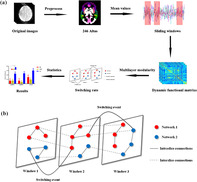

## 1 INTRODUCTION

The clinical identification of BD patients under depressive state from MDD patients on the basis of symptomology only is a challenge (Kamat et al., 2008). The BD misdiagnosis rate is up to 69% (Hirschfeld, Lewis, & Vornik, 2003), resulting in improper treatment, high medical costs, and poor outcomes (Hirschfeld et al., 2003; Keck, Kessler, & Ross, 2008). Nowadays, researchers begin to focus their attention on finding robust biological markers with functional magnetic resonance imaging (fMRI) to distinguish BD from MDD.

Depression is hypothesized to be linked to deficient adaptive functions (Ellard et al., 2018). Resting state functional networks, such as default mode network (DMN) and salience network (SN), are found to be linked to key adaptive functions including emotion regulation, attentional control, and self‐monitoring (Seeley et al., 2007; Yeo et al., 2011). Deficits in these networks lead to dysfunction of emotion and attention across mood states in depression (Sheppes, Suri, & Gross, 2015). For example, DMN is in charge of self‐monitoring (Yeo et al., 2011), and deficit in DMN results in rumination in depression (Eshel & Roiser, 2010; Pizzagalli, Dan, Hallett, Ratner, & Fava, 2009). SN plays a vital role in emotional control, processing of interoceptive states and modulating network switching between DMN and central executive network (CEN) (Devarajan, Levitin, & Vinod, 2008; Goulden et al., 2014; Terasawa, Fukushima, & Umeda, 2013). Overreliance upon endogenous cues resulted from altered SN is related to reduced motivation to interact with external environment in depression (Nolen‐Hoeksema, Wisco, & Lyubomirsky, 2008). What is more, recent findings hint that adaptive processes might be differently altered in BD and MDD patients (Ellard et al., 2018). For example, the functional connectivity of the anterior insula and amygdala distinguishes BD from MDD patients (Ambrosi et al., 2017; Sheppes et al., 2015), suggesting distinctly pathophysiological mechanisms of emotional dysfunction (Ambrosi et al., 2017). When recalling autobiographical memory, the activity amygdala in BD patients is different from that in MDD patients (Young, Bodurka, & Drevets, 2016). BD and MD patients exhibit robust group specific alterations in large‐scale brain networks (Goya‐Maldonado et al., 2016), especially alteration of key hubs in adaptive functions (Ellard et al., 2018). However, previous studies ignore the dynamics of the brain function and do not directly explore dynamic networks configuration that is needed in adaptive regulation.

Investigation of network dynamics is one of the emerging frontiers of network neuroscience (Bassett & Sporns, 2017). The brain function is dynamic even in the resting state (Hellyer et al., 2014; Sakoğlu et al., 2010). The dynamic exchange of information occurs within and between networks (Hutchison, Thilo, Gati, Stefan, & Menon, 2013), along with networks configuration (Allen et al., 2014; Xiao & Duyn, 2013). These time varying interactions enable the brain to dynamically integrate and coordinate different neural systems in response to internal and external stimuli (Hutchison et al., 2013). Investigating dynamics of the brain function provides subtle insight into dynamic network reconstruction and temporal evolution of the human brain in various neurological and psychiatric conditions (Avena‐Koenigsberger, Misic, & Sporns, 2017; Calhoun, Miller, Pearlson, & Adalı, 2014). However, it is a challenge to explore dynamic networks configuration. A novel method named multilayer network analysis offers the possibility of detecting the dynamic networks configuration across time and space. On the basis of the algorithm, the time‐resolved fMRI functional connectivity is divided into non‐overlapping modules spanning time and space (Mucha, Thomas, Kevin, Porter, & Jukka‐Pekka, 2010). Based on this method, Bassett et al propose node flexibility (or network switching rate [Pedersen, Zalesky, Omidvarnia, & Jackson, 2018]) defined as the percentage of time windows when the node changes to different network assignments (Bassett et al., 2011). Network switching rate is found to be related with learning (Bassett et al., 2011), attention (Shine, Koyejo, & Poldrack, 2016), fatigue (Betzel, Satterthwaite, Gold, & Bassett, 2017), depression (Zheng et al., 2017), and even predicting high‐order cognitive functions (Pedersen, Zalesky, et al., 2018). These studies affirm the underlying neurobiological basis of network switching resulted from networks configuration during adaptive functions.

In the current study, we aimed to explore distinct deficits in the adaptive functions in BD and MDD patients. We first constructed dynamic network matrixes (N × N × W for each subject, where N meant the regions in brain atlas in the current study and W meant the number of sliding windows) using a sliding windows method (Allen et al., 2014), then tracked (Mucha et al., 2010) the dynamic networks configuration overtime. Finally, the network switching rate of each node was calculated to measure its flexibility to exchange during networks configuration. On the basis of previous studies, we hypothesized that: (a) BD and MDD patients shared deficits in network switching rate of regions that were responsible for the common clinical symptoms, such as rumination. (b) Considering the fact that BD and MDD patients presented distinctly altered emotion control (Ambrosi et al., 2017; Sheppes et al., 2015), regulatory control (Ellard et al., 2018), and reward processing (Redlich et al., 2015; Whitton, Treadway, & Pizzagalli, 2015), the network switching rate of the key hubs in charge of these processes might be differently altered in BD and MDD patients.

## 2 MATERIALS AND METHODS

### 2.1 Participants

Forty BD and 61 MDD patients were recruited from the Clinical Hospital of Chengdu Brain Science Institute, University of Electronic Science and Technology of China. All patients were interviewed by two experienced psychiatrists using the Structured Clinical Interview for DSM‐IV‐TR‐Patient Edition (SCID‐P, 2/2001 revision) and diagnosed with BD or MDD on the basis of the DSM‐IV criteria. The 24‐Item Hamilton Depression Scale was used to evaluate the clinical state of the patients and all patients were under depressive state. Patients were excluded if they met one of the exclusion criteria. The exclusion criteria included schizophrenia, mental retardation, personality disorder, any history of loss of consciousness, substance abuse, and serious medical or neurological illness. In the current study, the patients were excluded if they were diagnosed with anxiety disorder at same time. MDD patients were treated with antidepressants. The drugs administered included one of the selective serotonin and serotonin–norepinephrine reuptake inhibitors. BD patients were treated with antidepressants, mood stabilizer, and antipsychotics.

Considering that most of patients were taking medicine, to measure the effect of medicine on our results, we used a strategy named the total medication load (Redlich et al., 2014; Redlich et al., 2015). As done in previous studies, the antidepressant, mood stabilizer, and antipsychotic medication was first coded as absent (0), low (1), or high (2). The total medicine load was calculated by summing each medication code for each medication category. The details could be seen in our previous work (Han et al., 2018).

Sixty‐three healthy controls (HCs) were recruited from the community through poster advertisements and also interviewed using SCID (nonpatient edition). HCs were matched in terms of age, gender, and years of education with the patient groups. No healthy participant had a history of serious medical or neuropsychiatric illness and a family history of major psychiatric or neurological illness in their first‐degree relatives.

Written informed consents were obtained from all participants before the experiment. The study was approved by the research ethical committee of the University of Electronic Science and Technology of China.

### 2.2 Scan acquisition

All MRI data was acquired with a 3‐T GE Discovery MR750 scanner (General Electric, Fairfield, CN). An eight‐channel prototype quadrature birdcage head coil fitted with foam padding to minimize head movement was used. All subjects were instructed to relax, to hold still, and to keep their eyes closed during the scan. Functional images were obtained using an echo‐planar imaging sequence with the following parameters: TR/TE = 2,000/30 ms, 43 slices, matrix size = 64 × 64, voxel size = 3.75 × 3.75 × 3.2 mm^3^, flip angle = 90°, slice thickness = 3.2 mm, no gap, and total 255 volumes.

### 2.3 Data preprocessing

Functional images were preprocessed using Data Processing Assistant for Resting‐State fMRI package (http://www.restfmri.net). The following steps were performed in preprocessing stage: (a) removal of the first 15 volumes; (b) slice timing; (c) realignment; (d) normalization to the standard EPI template (resampled into 3 × 3 × 3 mm^3^), subjects were excluded if the translational and rotational displacement exceeded 3.0 mm or 3.0°. No subject was excluded in this step: (e) smoothing (6 × 6 × 6 mm^3^ full‐width at half maximum Gaussian kernel); (f) detrend to reduce low frequency drift; (g) regression of nuisance covariates, including Friston 24 motion parameters (Satterthwaite et al., 2012), global mean signal, white matter signal, and cerebrospinal fluid signal; and (h) despiking. We replaced the outliners with the optimal estimate using a third‐order spline fit to clean the time course portions. Outliners were detected on the basis of the median absolute deviation, as implemented in 3dDespike (http://afni.nimh.nih.gov/afni) (Allen et al., 2014). We also calculated the mean frame‐wise displacement (FD) of each subject (Han et al., 2017). No significant difference of mean FD was observed among the three groups by using ANOVA (*p* = .60).

### 2.4 Dynamic functional connectivity with a sliding window method

**FIGURE 1:**
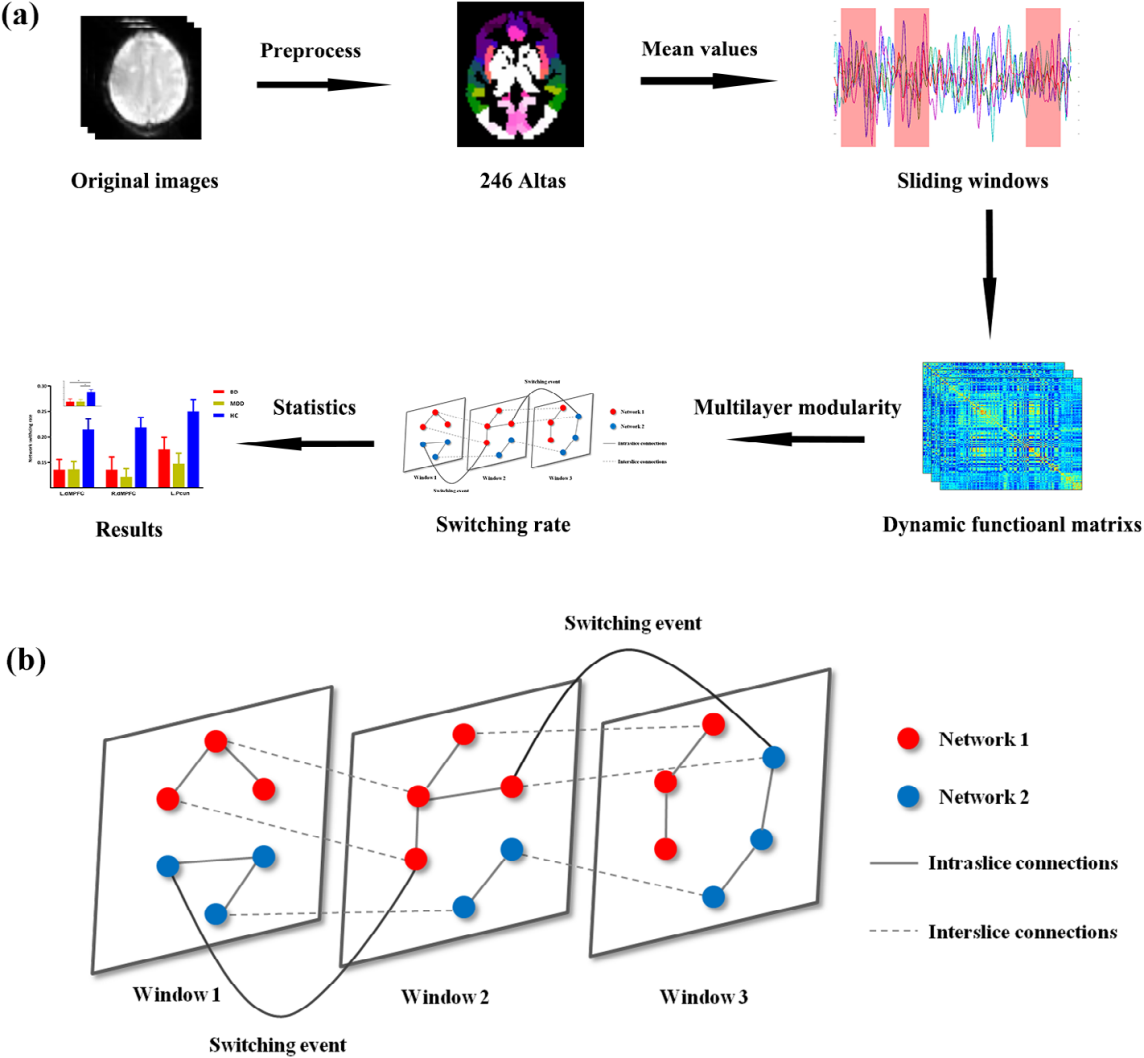
(a) Flow chart of analysis steps. The original images were preprocessed. The mean values of 246 atlas were extracted to build dynamic functional matrix for each subject. Then, an iterative and ordinal Louvain algorithm was used to track dynamic network modulation over time. Finally, network switching rate was calculated and compared among three groups with ANOVA. (b) An overview of network switching rate. The windows meant the sliding windows in dynamic functional connectivity. The red and blue nodes represented nodes in two different modularity partitions. There were two switching events in this figure (adapted from Mangor et al.)

The averaged signals of each region in 246 atlas were extracted (Fan et al., 2016). A sliding windows method was adopted to calculate the dynamic functional connectivity (Allen et al., 2014) where the window length was set to 50 TRs (100 s) and step length was set to the half window length (25 TRs, 50 s) (Guo et al., 2018; Nora & Dimitri, 2015; Pedersen, Omidvarnia, Zalesky, & Jackson, 2018; Zalesky & Breakspear, 2015). Pearson correlation between pair of region signals in each window was calculated. The overall flow chat of analysis was show in Figure 1a. Finally, we would get dynamic networks matrixes (N × N × W) for each subject. Where N meant the regions in brain atlas in the current study and W meant the number of sliding windows.

To exclude the effect of parameter selection like window length and step size on our results, we also validated our results with different window length (window length = 22 TRs, step size = 11 TRs).

### 2.5 Multilayer modularity and network switching rate

As performed in previous studies (Bassett et al., 2011; Bassett et al., 2013; Pedersen, Zalesky, et al., 2018), an iterative and ordinal Louvain algorithm was used to track dynamic networks configuration over time (Mucha et al., 2010) where parameters were set *ω* = *ϒ* = 1 (Bassett et al., 2013; Pedersen, Zalesky, et al., 2018). This multilayer modularity algorithm outputted a 2D array with integer values indicating modules and *Q*. A higher *Q*, ranging from zero to one, meant higher network segregation. By now, the dynamic matrixes for each subject were divided into nonoverlapping modules spanning time and space (Mucha et al., 2010). Then the switching rate of each node was calculated as the percentage of time windows when the node changed to a different network assignment (Telesford et al., 2017) (Figure 1b).

### 2.6 Statistical analysis

One‐way ANOVA was conducted among the three groups to determine the altered network switching rate of nodes and overall dynamic networks segregation (*Q*) in MDD and BD patients. In this step, sex, age, and years of education were regressed as covariates. The results of altered network switching rate (for 246 nodes in all) reported here were corrected with false discovery rate (FDR), where *p* < .05. Then, to explore details about aberrance in BD or MDD, two tailed two sample *t* test was adopted between BD (or MDD) patients and HCs for nodes whose network switching rate presenting differences among three groups.

### 2.7 Validation

Considering the effect of window length, we validated our results with window length of 22 TRs and step size of 11 TRs.

To exclude the effect of medicine (or head motion) on observed results, Pearson correlation was calculated between the altered network switching rate of nodes with the total medicine load (or mean FD) with network switching rate of nodes (or *Q*) in MDD (or BD) patients. In addition, the mean FD was compared among three groups and between BD and MDD patients.

## 3 RESULTS

### 3.1 Clinical effects

To determine whether the total sample size was enough in the current study, we done a priori power analysis with G power 3 (Faul, Erdfelder, Lang, & Buchner, 2007) (significant level *α* = .05, power level = 0.85 or 0.8 and effect size *f* = 0.25). The total sample size was computed as 180 or 159 (the total sample size in the current study was 164) suggesting the sample size used was basically enough. No significant difference was found among groups in terms of sociodemographic characteristics, such as age, gender, and year of education. The medicine load index of MDD was lower than that in BD (*p* < .01). Details could be seen in Table 1.

**TABLE 1:** Characteristics of subjects

	BD (*n* = 40)	MDD (*n* = 61)	HC (*n* = 63)	*p*
Age (years), mean ± *SD*	34.43 ± 10.76	34.56 ± 11.07	31.76 ± 10.50	.29^a^
Gender, male:female (percent of male)	18:22 (45.00%)	33:28 (54.10%)	33:30 (52.38%)	.65^b^
Years of education, mean ± *SD*	13.70 ± 3.03	13.56 ± 3.19	14.16 ± 3.88	.60^a^
Handedness, right/left	40/0	61/0	62/1	.45^a^
Age of first onset (years)	27.03 ± 9.57	29.67 ± 10.44	—	.20^c^
No. of depression episodes	2.63 ± 1.13	2.03 ± 0.98	—	<.01^c^
Duration of single depressive episode	3.93 ± 2.46	5.30 ± 6.70	—	.06^c^
HAMD	24.89 ± 5.28	22.93 ± 6.42	—	.10^c^
Medical medicine load index	3.13 ± 1.34	2.15 ± 0.79	—	<.01^c^
Percentage of taking medicine	92.50%	98.36%	—	—
Antidepressants (number of patients)				
Fluoxetine	3	2		
Sertraline	10	10		
Paroxetine	7	19		
Escitalopram	5	10		
Fluvoxamine	0	0		
Venlafaxine	3	8		
Duloxetine	1	5		
Mirtazapine	0	2		
Mood stabilizer				
Valproate	27	0		
Lamotrigine	2	0		
Lithium	5	0		
Antipsychotics				
Olanzapine	9	6		
Quetiapine	19	10		
Risperidone	2	2		
Aripiprazole	2	1		

Abbreviations: BD, bipolar disorder; HC, healthy control; MDD, major depressive disorder.

a One‐way ANOVA.

b Chi‐square *t* test.

c Two‐tailed two sample *t* test.

### 3.2 Network switching rate is decreased differently in BD and MDD patients

The overall modularity (*Q*) significantly differed among the three groups (*p* = .046 for ANOVA). Specially, the *Q* significantly decreased in MDD patients compared with HCs (*p* = .018 for two sample *t* test). There was no significant difference between BD patients and HCs (Figure S1).

The three groups presented significantly different network switching rate of regions in DMN (left precuneus, bilateral parahippocampal gyrus, and bilateral dorsal medial prefrontal cortex [dMPFC]), SN (bilateral anterior insula and left amygdala) and the left striatum (*p* < .05, FDR corrected).

**FIGURE 2:**
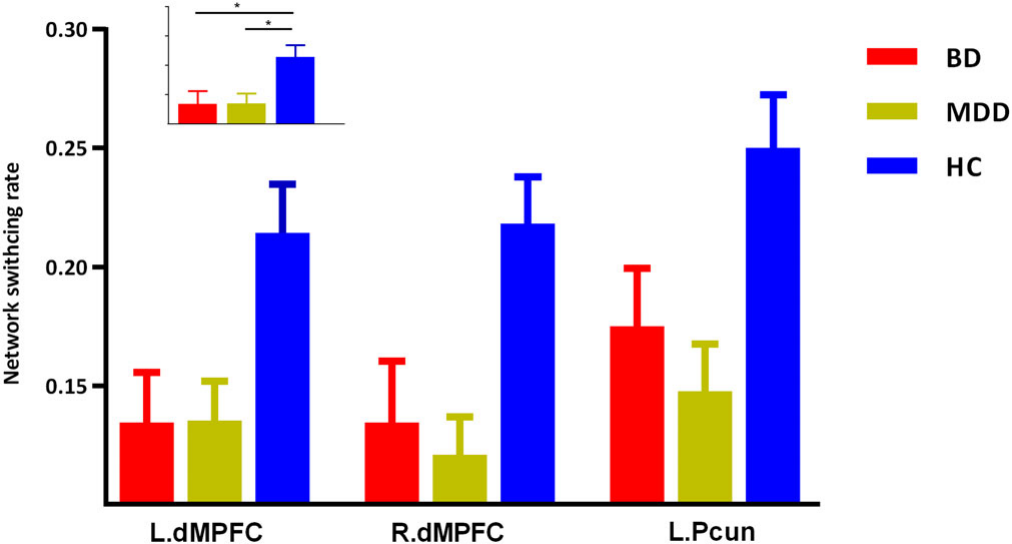
Shared decreased network switching rate of BD and MDD patients. L.dMPFC, left dorsal medial prefrontal cortex; L.Pcun, left precuneus

**FIGURE 3:**
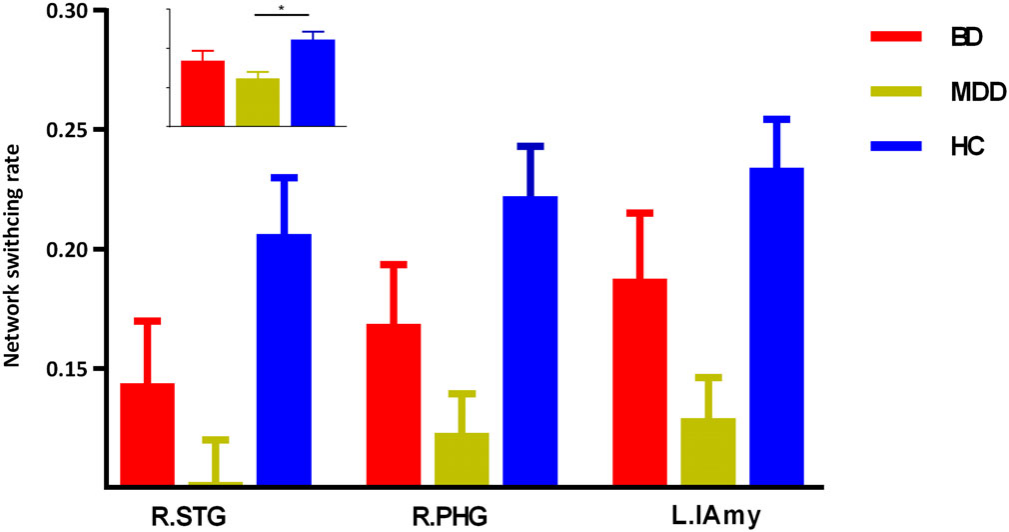
Specially decreased networking rate in MDD patients. L.lAmy, left lateral amygdale; R.STG, right superior temporal gyrus; R.PHG, right parahippocampal gyrus

**FIGURE 4:**
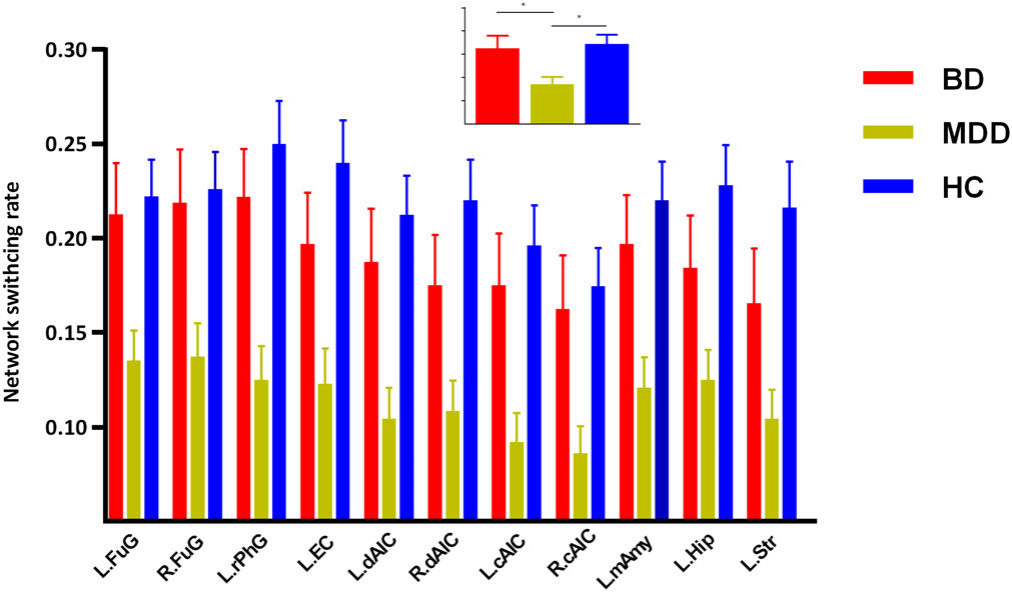
Regions presenting lower network switching rate in MDD patients compared with BD patient. L.Fug, fusiform gyrus; L.rPhG, left rostral parahippocampal gyrus; L.EC, left entorhinal cortex; L.dAIC, left dorsal anterior insula cortex; L.mAmy, left medial amygdale; L.Hip, left hippocampus; L.str, left stritum; R.cAIC, right caudoventral anterior insula cortex

The post hoc results demonstrated that both BD and MDD patients presented decreased network switching rate of regions in DMN (left precuneus and bilateral dMPFC) (Figure 2). While, MDD patients also presented decreased network switching rate in SN, and left striatum (Figures 3 and 4). What is more, MDD patients presented significantly lower network switching rate in SN and left striatum compared with BD patients (Figure 3). The details were showed in Table S1.

### 3.3 Validation

Our results reported above could be validated with different window length and step size (Table S2). There was no significant difference of mean FD among three groups (*p* = .60 for ANOVA). The mean FD in BD patients was not significantly different from that in MDD patients (*p* = .35 for two sample *t* test). The correlations between medicine load (and mean FD) with altered network switching rate of nodes were all not significant (*p* > .05).

## 4 DISCUSSION

In the current study, we explored distinctly altered dynamic networks configuration with network switching rate in BD and MDD patients. As we assumed, BD and MDD patients shared decreased network switching rate in the key hubs of DMN, including left precuneus and bilateral dMPFC. These results suggested shared inability to navigate away from internal emotional and cognitive states across depressed BD and MD patients. Apart from these regions, MDD patients also presented a decrease in network switching rate of SN and striatum. Moreover, network switching rate of SN and striatum in MDD patients was significantly lower than that in BD patients. These results might reflect special deficient switching ability and emotion regulation in MDD patients. We found shared and distinct aberrance of network flexibility which revealed altered adaptive functions during dynamic networks configuration of BD and MDD.

### 4.1 BD and MDD patients shared decreased network switching rate in DMN

In our results, BD and MDD patients shared decreased network switching rate in DMN. The decreased network switching rate of MDD might reflect inability to navigate away from internal emotional and cognitive states in depression (Greicius et al., 2007; Whitfieldgabrieli & Ford, 2012).The DMN was in charge of various functions, such as self‐referential thinking, remembering the past, and focusing on beliefs (Buckner & Vincent, 2007; Raichle & Snyder, 2007). Dysfunction of DMN was found across BD and MDD patients (Martino et al., 2016; Meda et al., 2014; Mulders, van Eijndhoven, Schene, Beckmann, & Tendolkar, 2015). For example, MDD patients presented reduced DMN task suppression during emotion perception and judgment (Grimm et al., 2009) and during passive viewing and reappraisal of negative pictures (Sheline et al., 2009). The hyperactivity in task performance and increased connectivity of DMN were found in depressed subjects, this might reflect the inability to navigate away from their internal emotional and cognitive states (such as, rumination) (Greicius et al., 2007; Whitfieldgabrieli & Ford, 2012). The excessive DMN functional connectivity could be seen even in recovered‐state MDD patients (Nixon et al., 2013). The hyperactivity of DMN might be resulted from incapable suppression of DMN (Hamilton et al., 2011). The under activation and reduced global brain connectivity of dMPFC, a key region in emotion regulation (Cole & Schneider, 2007; Ochsner & Gross, 2005), were found in depressed patients (Murrough et al., 2016; Siegle, Wesley, Carter, Steinhauer, & Thase, 2007). In line with these findings, we observed decreased network switching rate of regions in DMN in BD and MDD. These results might reflect reduced cognitive flexibility and reduced emotion regulation capacity associated with depressed patients (Murrough et al., 2016; Murrough, Iacoviello, Neumeister, Charney, & Dan, 2011).

### 4.2 MDD patients presented decreased network switching rate in SN compared with HCs

Apart from DMN, the network switching rate of striatum and key hubs in SN was also decreased in MDD patients. The anterior insula was linked to the processing of interoceptive states and adaptive switching between DMN and CEN (Devarajan et al., 2008; Terasawa et al., 2013). The important role of the anterior insula in mapping the visceral autonomic aspects of emotional experience to a high‐order cognitive process (Craig, 2003) made it a key structure in integrating cognitive, behavioral, and affective functional processes (Devarajan et al., 2008; Goulden et al., 2014). The anterior insula was a region of particular interest in mood disorders (Goodkind et al., 2015). Overreliance upon endogenous cues was related to reduced motivation to interact with external environment in MDD (Nolen‐Hoeksema et al., 2008). The functional connectivity between the anterior insula and DMN was found increased in MDD patients compared with BD patients and HCs (Ellard et al., 2018). The decreased network switching rate of the anterior insula might reflect its impaired switching ability to instantiate regulation away from international, self‐focused processing in MDD (Ellard et al., 2018). The amygdala was involved in emotion regulation and sensory information modulation (Price, 2010) and essential for sensory tuning to enable adaptive response (Davis & Whalen, 2001). The impaired flexibly switching of the amygdala might be relevant to emotion dysregulation and attention (cognitive) bias observed in MDD patients (Albert & Newhouse, 2019). The decreased network switching rate hinted decreased ability in adaptive emotion regulation and ability to instantiate regulation away from internal, self‐focused processing (Ellard et al., 2018).

### 4.3 MDD patients presented decreased network switching rate in SN and striatum compared with BD

The network switching rate of regions in SN (anterior insula and amygdala) and striatum, was significantly lower in MDD patients compared with BD patients and HCs. Previous studies found structural and functional anomalies of these regions (SN and striatum) in BD patients, suggesting altered emotion regulation and reward processes (Albert & Newhouse, 2019; Ambrosi et al., 2017; Ellard et al., 2018; Han, De Berardis, Fornaro, & Kim, 2018; Satterthwaite et al., 2015). We found no significantly altered network switching rate of these regions in BD patients compared with HCs. The reason might be that dynamic methods provide distinct insight into neuromechanism of depression (Han et al., 2019; Kaiser et al., 2015). However, consistent with previous studies (Albert & Newhouse, 2019; Ambrosi et al., 2017; Ellard et al., 2018), the network switching ability of these regions was significant different between BD and MDD patients. Combing previous findings, our results provided new insight into the different neural pathogenesis between MDD and BD. Dysfunction of these regions in BD patients might be more related to deficits in steady coordination with other regions (Albert & Newhouse, 2019; Ambrosi et al., 2017; Ellard et al., 2018) while that in MDD patients might be related to deficits in dynamic regulation capacity. Although this hypothesis required sufficiently direct evidences, our results affirmed different dysfunction of these regions between BD and MDD patients.

We excluded the effect of head motion and medicine on our results to a certain extent. In our results, no significant difference of the mean FD was found among the three groups. In addition, network switching rate of altered regions was not significantly correlated with mean FD. Our findings were unlikely resulted from the medicinal effect. Although antidepressant treatment may affect the functions of several regions, such as the prefrontal cortex and hippocampus, the conclusion was inconsistent (Hibar et al., 2016; Siegle et al., 2007). Dysfunction of these regions was affirmed with unmedicated patients (Cao et al., 2012; Geng et al., 2016; Murrough et al., 2016). Here, we found no significant correlation between network switching rate of altered regions and medicinal load.

A number of limitations must be considered in the current study. First, most patients (BD and MDD) were taking medicine. Although no significant correlation was observed between medicinal load and altered network switching rate in patients, our results should be validated by future studies in the sample of unmedicated patients. Second, patients (BD and MDD) enrolled in our study were under depressive state. The network switching rate might be differently altered in various mood states, such as remitted state (Rive et al., 2015). This hypothesis could be tested in future studies. Third, factors like illness duration which was different in BD and MDD patients, might also affect our results. Last, different aberrance of the network switching rate might also due to many phenotypes shared between BD and MDD but that were not specific to these two disorders, such as chronic stress, poor sleep, feelings of stigma, and so on. Because these phenotypes might be shared with basically any other psychiatry disorder, future studies could recruit other psychiatry disorders to explore specific altered dynamic switching ability across psychiatry disorders.

## 5 CONCLUSION

We explored distinct deficits in dynamic switching ability in BD and MDD using multilayer network analysis. BD and MDD patients shared decreased network switching ability in DMN. MDD patients presented specially and discriminately decreased network flexibility of SN and striatum compared with BD patients and HCs. Our results suggested shared and distinct aberrance of adaptive functions during dynamic networks configuration of BD and MDD.

## CONFLICT OF INTEREST

All authors declared no conflict of interest.

## Supporting information


**Figure S1** Decreased *Q* in MDD patients.
**Table S1** Results of post hoc analysis, all *p* values were original *p* values.
**Table S2** Validated results with a window length of 22 TRs and step size of 11 TRs.

## Data Availability

Data sharing is not applicable to this article as no new data were created or analyzed in this study.
