# Supplementary material for: Resting state functional network switching rate is differently altered in bipolar disorder and major depressive disorder
Source: Hum Brain Mapp. 2020 May 13;41(12):3295–304. doi: 10.1002/hbm.25017 (PMC7375077; doi:10.1002/hbm.25017)
Supplement: Supplementary file 1 — Figure S1 Decreased Q in MDD patients. Table S1 Results of post hoc analysis, all p values were original p values. Table S2 Validated results with a window length of 22 TRs and step size of 11 TRs. [file HBM-41-3295-s001.docx]

**sFigure 1**. Decreased Q in MDD patients.





**sTable1**.Results of post hoc analysis, All *p* values were original *p* values.

| Regions | BD < HC (t, *p*) | MDD < HC (t, *p*) | BD > MDD (t, *p*) |
| --- | --- | --- | --- |
| L.dMPFC | -2.598, 0.011 | -2.987, 0.003 | -0.033, 0.974 |
| R.dMPFC | -2.615, 0.010 | -3.836, <0.001 | 0.468, 0.341 |
| L.Pcun | -1.737, 0.031 | -3.516, <0.001 | 1.365, 0.387 |
| R.STG | -0.297, 0.086 | -3.454, <0.001 | 2.621, 0.175 |
| R.PHG | -0.224, 0.106 | -3.363, <0.001 | 2.577, 0.111 |
| L.lAmy | -0.801, 0.169 | -4.305, <0.001 | 3.215, 0.060 |
| L.Fug | -1.631, 0.767 | -3.725, <0.001 | 1.609, 0.010 |
| R.Fug | -1.214, 0.824 | -3.999, <0.001 | 2.325, 0.011 |
| L.rPhG | -2.189, 0.106 | -3.406, <0.001 | 0.870, 0.111 |
| L.EC | -0.717, 0.228 | -4.046, <0.001 | 2.721, 0.221 |
| L.dAIC | -1.316, 0.475 | -4.135, <0.001 | 2.256, 0.008 |
| R.dAIC | -0.623, 0.191 | -3.988, <0.001 | 2.847, 0.026 |
| L.cAIC | -0.355,0.535 | -3.531, <0.001 | 2.628, 0.005 |
| R.cAIC | -0.706, 0.723 | -3.800, <0.001 | 2.630, 0.010 |
| L.mAmy | -1.386, 0.482 | -3.952, <0.001 | 1.905, 0.010 |
| L.Hip | -1.274, 0.206 | -3.880, <0.001 | 1.992, 0.049 |
| L.str | -1.325, 0.188 | -3.861, <0.001 | 2.042, 0.044 |

Note: L.dMPFC, left dorsal medial prefrontal cortex; R.dMPFC, right dorsal medial prefrontal cortex; L.Pcun, left precuneus; R.STG, right superior temporal gyrus; R.PHG, right parahippocampal Gyrus; L.lAmy, left lateral maydala; L.Fug, fusiform gyrus; L.rPhG, left rostral parahippocampal gyrus; L.EC, left entorhinal cortex; L.dAIC, left dorsal anterior insula cortex; R.cAIC, right caudoventral anterior insula cortex; L.mAmy, left medial amygdale; L.Hip, left hippocampus; L.str, left stritum.

**sTable2**. Validated results with with a window length of 22 TRs and step size of 11 TRs.

| Regions | *P* for ANOVA |
| --- | --- |
| L.dMPFC | 0.003 |
| R.dMPFC | <0.001 |
| L.Pcun | 0.002 |
| R.STG | 0.003 |
| R.PHG | 0.003 |
| L.lAmy | <0.001 |
| L.Fug | 0.002 |
| R.Fug | <0.001 |
| L.rPhG | 0.002 |
| L.EC | <0.001 |
| L.dAIC | <0.001 |
| R.dAIC | <0.001 |
| L.cAIC | 0.003 |
| R.cAIC | 0.001 |
| L.mAmy | 0.001 |
| L.Hip | 0.001 |
| L.str | 0.001 |

Note: L.dMPFC, left dorsal medial prefrontal cortex; R.dMPFC, right dorsal medial prefrontal cortex; L.Pcun, left precuneus; R.STG, right superior temporal gyrus; R.PHG, right parahippocampal Gyrus; L.lAmy, left lateral maydala; L.Fug, fusiform gyrus; L.rPhG, left rostral parahippocampal gyrus; L.EC, left entorhinal cortex; L.dAIC, left dorsal anterior insula cortex; R.cAIC, right caudoventral anterior insula cortex; L.mAmy, left medial amygdale; L.Hip, left hippocampus; L.str, left stritum.
